# Epidemiology of *Mansonella perstans* in the middle belt of Ghana

**DOI:** 10.1186/s13071-016-1960-0

**Published:** 2017-01-07

**Authors:** Linda Batsa Debrah, Norman Nausch, Vera Serwaa Opoku, Wellington Owusu, Yusif Mubarik, Daniel Antwi Berko, Samuel Wanji, Laura E. Layland, Achim Hoerauf, Marc Jacobsen, Alexander Yaw Debrah, Richard O. Phillips

**Affiliations:** 1 Kumasi Centre for Collaborative Research into Tropical Medicine (KCCR), Kumasi, Ghana; 2 Department of General Pediatrics, Neonatology, and Pediatric Cardiology, University Children’s Hospital, Duesseldorf, Germany; 3 Institute for Medical Microbiology, Immunology and Parasitology, University Hospital of Bonn, Bonn, Germany; 4 Faculty of Allied Health Sciences of Kwame Nkrumah University of Science and Technology, Kumasi, Ghana; 5 Department of Medicine, Kwame Nkrumah University of Science and Technology, Kumasi, Ghana; 6 Department of Microbiology and Parasitology, University of Buea, Buea, Cameroon; 7 Department of Clinical Microbiology, Kwame Nkrumah University of Science and Technology, Kumasi, Ghana

**Keywords:** Mansonellosis, *Mansonella perstans*, *Culicoides*, Microfilariae, Epidemiology

## Abstract

**Background:**

Mansonellosis was first reported in Ghana by Awadzi in the 1990s. Co-infections of *Mansonella perstans* have also been reported in a small cohort of patients with Buruli ulcer and their contacts. However, no study has assessed the exact prevalence of the disease in a larger study population. This study therefore aimed to find out the prevalence of *M. perstans* infection in some districts in Ghana and to determine the diversity of *Culicoides* that could be potential vectors for transmission.

**Methods:**

From each participant screened in the Asante Akim North (Ashanti Region), Sene West and Atebubu Amantin (Brong Ahafo Region) districts, a total of 70 μl of finger prick blood was collected for assessment of *M. perstans* microfilariae. Centre for Disease Control (CDC) light traps as well as the Human Landing Catch (HLC) method were used to assess the species diversity of *Culicoides* present in the study communities.

**Results:**

From 2,247 participants, an overall prevalence of 32% was recorded although up to 75% prevalence was demonstrated in some of the communities. *Culicoides inornatipennis* was the only species of *Culicoides* caught with the HLC method. By contrast, *C. imicola* (47%), *C. neavei* (25%) and *C. schultzei* (15%) were caught by the CDC light trap method. A wide diversity of other *Culicoides* spp. was also identified but correlation was only found between the prevalence of *C. inornatipennis* and *M. perstans* during the dry season.

**Conclusions:**

Here we demonstrate for the first time that *M. perstans* is highly prevalent in three districts in Ghana. We found a wide spectrum of *Culicoides* spp. *Culicoides inornatipennis* was the most anthropophilic and is therefore likely to be the species responsible for transmission of infection but formal proof has yet to be obtained.

**Trial registration:**

NCT02281643. Registered October 26, 2014. ‘Retrospectively registered’. Trial Registry: ClinicalTrials.gov.

**Electronic supplementary material:**

The online version of this article (doi:10.1186/s13071-016-1960-0) contains supplementary material, which is available to authorized users.

## Background

Amongst the known human filarial infections that parasitize man, mansonellosis is the least studied even though more than 100 million individuals are estimated to be infected [1]. Prevalence of mansonellosis has mainly been reported in sub-Saharan Africa and parts of Central and South America with about 600 million individuals being at risk of infection in about 33 countries [1, 2]. To date, mansonellosis in man is elicited by three known agents: *M. perstans*, *M. streptocerca* and *M. ozzardi*. These agents vary in morphological and anatomical features as well as in their geographical locations [3]. Whereas *M. perstans* and *M. streptocerca* occur mainly in western, eastern and central Africa, *M. ozzardi* is only prevalent in South America [3].

As with other filariae*, M. perstans* requires a vector and is transmitted by female flies of the genus *Culicoides* (biting midges). The overall life-cycle is similar to those of other nematode infections such as onchocerciasis, lymphatic filariasis and loiasis where humans are the definitive host. In brief, infective larvae are introduced into the host during a blood meal and develop into adult worms that reside in the coelomic cavity, peritoneal and pleural cavities as well as mesenteric perirenal and retroperitoneal tissues [4]. They produce thousands of unsheathed microfilariae (MF) that appear in the blood stream with no particular periodicity and are taken up by another biting midge for transmission to continue [1]. The exact lifespan of *M. perstans* adult worms has not been deciphered although there is a report of the presence of MF in the blood of a person who left the endemic community 10 years earlier [5].

Mansonellosis has previously been thought of as non-pathogenic [1, 6] but there have been associations of some clinical manifestations with MF release, such as eosinophilia and ocular disorders [7–9]. Other studies have implicated *M. perstans* in the disease progression of clinical malaria [10] and co-infections have been shown to influence infant morbidity in Uganda [11–13]. In Ghana, *M. perstans* was detected in the 1990s [14] but its relevance was not documented until its association with *Mycobacterium ulcerans* was noted in Buruli ulcer endemic communities in the Asante Akim North District [15]. Epidemiological surveys on *M. perstans* prevalence in Ghana are lacking and the distribution of different *Culicoides* species serving as potential vectors is not known.

Thus given the paucity of information regarding *M. perstans* prevalence in Ghana and also the existence of different species of vector(s) involved in the transmission of *M. perstans* in Africa [16–18], we carried out epidemiological studies of *M. perstans* to assess the burden of infection and to further identify the diversity of *Culicoides* species that are attracted by light and humans.

## Methods

### Study design

**Fig. 1:**
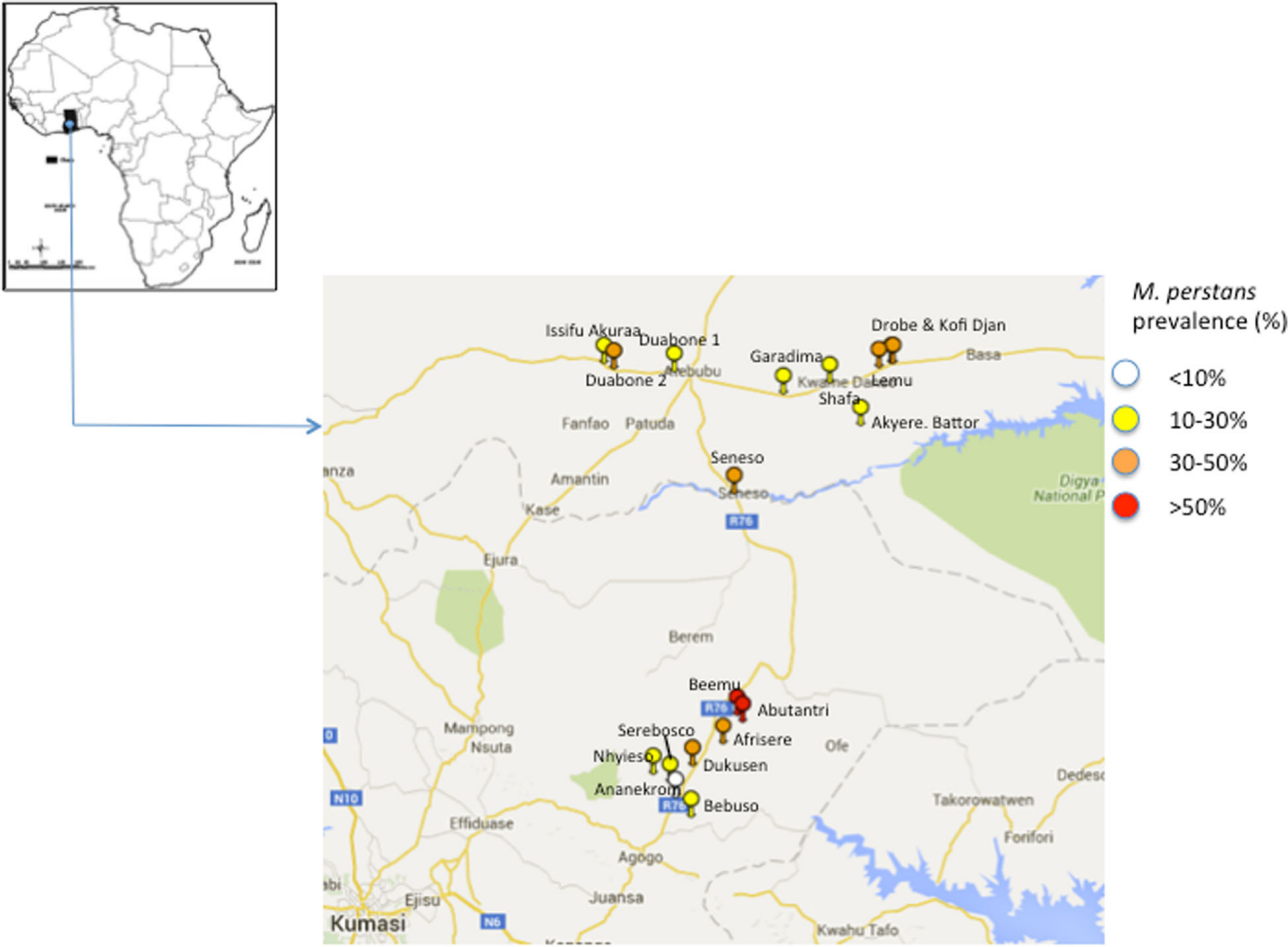
Spatial-epidemiological map of study communities

*Mansonella perstans* prevalence was determined in 2,247 participants from areas of the Middle Belt of Ghana between July 2014 and September 2015. From the Ashanti Region, eight communities (Sereboso, Nhyieso, Dukusen, Beemu, Bebuso, Ananekrom, Afrisere and Abutantri) were selected in the Ashanti Akim North District based on previous reports of the disease [15]. From the Brong Ahafo region, five communities (Akyeremade Battor, Drobe, Kofi Gyan, Lemu and Shafa) in the Sene West and five communities (Duabone No. 1, Duabone No. 2, Garadima, Issifu Akuraa and Seneso) in the Atebubu-Amantin Districts were also selected due to considerable migration of people from the Ashanti Akim North District to these communities. These three districts are in tropical rainforest areas with similar ecological characteristics. Entomological surveys were also performed in communities in the Ashanti Akim North District to determine diversity of *Culicoides* species that are attracted to light and/or humans (Fig. 1).

General demographic information such as age, sex, and history of previous Mass Drug Administration (MDA) were obtained from the consented participants. A total of 70 μl finger pricked blood samples was taken under sterile conditions for parasitological assessment. Entomological assessment of *Culicoides* spp. was carried out during the same period.

### Parasitological examination

#### Microfilariae assessment by direct finger pricking

The middle finger of the volunteer’s left hand was wiped and massaged simultaneously to ensure adequate blood flow to the tip of the finger. The skin of the tip of the finger was disinfected with 70% alcohol, pricked with a sterile lancet and 20 μl of blood pipetted onto a clean-labelled microscope slide. A coverslip was placed on the blood and directly observed under 10× objective lens of the light microscope for the presence of motile microfilariae.

#### Microfilariae assessment by counting chamber technique

To confirm that the MF identified in the direct finger prick blood were *M. perstans*, 50 μl of blood was additionally transferred from the pricked finger into already prepared 1.5 ml Eppendorf tubes containing 950 μl of 3% acetic acid and mixed thoroughly to haemolyse the red cells. The solution was poured into the Sedgewick counting chamber ensuring the absence of air bubbles. The presence or absence of *M. perstans* MF was assessed under a light microscope using the 10× objective lens. The MF were identified morphologically as *M. perstans* based on the absence of sheath, extension of nuclei to the bluntly rounded tip and their small size (usually about 200 μm).

### Entomological survey

Seasonal collections of *Culicoides* species were carried out from June to September 2014 for the wet season and November 2014 to February 2015 for the dry season, which are the two main seasonal variations in Ghana. *Culicoides* species were collected during the first week of each month.

### Light trap collections

Centre for Disease Control’s (CDC) New Standard Miniature light traps from John W. Hock Company, Florida, USA, were mounted in proximity to both breeding sites and human habitations. Each study community was divided into four parts and a light trap mounted in each part for the trapping of the midges in the dry and wet seasons. The same collection sites were used at both sampling times. Attracted by UV light emitted by the trap, *Culicoides* spp. and other flies were trapped in a Petri plate containing 80% alcohol placed in the suspended trap. Catches were made from 6:00 to 9:00 h for the morning periods and from 16:00 to 17:00 h for the evening periods. The trap catches were removed at the end of the three hours collection and each trap assigned a unique number with respect to its position in the field. An overnight trap was additionally mounted from 18:00 to 6:00 h. The trapped flies were transferred into labelled 50 ml falcon tubes containing 80% alcohol and placed in a cold box for transportation to the laboratory. The number of flies caught by each trap (6:00–9:00 h, 16:00–17:00 h and 18:00–6:00 h) was recorded and the flies identified morphologically [19].

### Human landing catches

Volunteers from the various communities were given training on how to collect midges using the human landing catch method. They were then deployed to their various communities, and collection of *Culicoides* was supervised by a trained staff member to reduce bias.

Midges were captured daily by 4 trained collectors stationed near human habitations in the study communities. Blood-seeking female midges were collected using locally made aspirators when they were attempting to take a blood meal on the collectors. The catching times were made to coincide with that of the aforementioned light traps. The caught midges were aspirated and stored into labelled transparent plastic cups covered with a fine net and blocked with cotton wool at the base. Midges were knocked-down by freezing for few minutes and then transferred into a labelled eppendorf tube containing 80% ethanol. They were then transported to the laboratory for morphological identification on the species level.

### Data analysis

Descriptive statistics was used to obtain general descriptive information using the StatView®, Version 5 for Windows. Data were analysed using IBM SPSS statistics software version 22 and *P*-values less than 0.05 (*P* < 0.05) considered statistically significant. Prism 6 software was used for plotting the graphs from data generated. Geospatial maps were created using http://www.spatialepidemiology. Mann-Whitney U-tests were carried out for the determination of the differences in demographic information between males and females and Spearman’s correlation was used to check for correlation between *M. perstans* prevalence and *Culicoides* abundance.

## Results

### Characteristics of the study populations according to districts

A total of 2,247 participants from 18 communities in Ashanti Akim North District, Atebubu Amantin District and Sene West Districts was screened for the presence of *M. perstans* MF in their blood. Upon comparison of participating districts, there was no significant difference in gender or age distribution with the exception of participants from the Sene West District where the proportion of female volunteers was significantly higher than males (Table 1).

**Table 1:** Characterization of volunteers in the study districts

Characteristics	Sex	District				*P*-value
		Ashanti Akim North	Atebubu Amantin	Sene West	Total	
No. of screened		1,215	508	524	2,247	0.794
	M	601	253	251	1,105	
	F	614	255	273	1,142	
Mean age yrs (range)		27.89 (9–98)	30.69 (9–89)	31.70 (9–92)	29.41 (9–98)	0.788
	M	28.55 (9–98)	32.08 (9–89)	29.87 (9–91)^a^	29.66 (9–98)	
	F	27.24 (9–83)	29.30 (9–85)	33.37 (9–92)^a^	29.17 (9–92)	
MF positive		407	158	160	725	< 0.001
	M	233	89	78^b^	400	
	F	174	69	82^b^	325	
MF prevalence (%)		33	31	31		0.391
MDA		No	No	Yes^c^		

^a^Significant differences observed in Sene West District (Mann-Whitney *U*-Test, *U* = 29,539, *Z* = -2.727, *P* = 0.0064)

^b^No significant difference between MF positive and gender (Fisher’s exact test, *P* = 0.435)

^c^One community in the Sene West District had taken part in the national mass drug administration programme (MDA)

*Abbreviations*: M, male; F, female, MF, microfilarae

### Prevalence of *M. perstans* infection in study communities

**Fig. 2:**
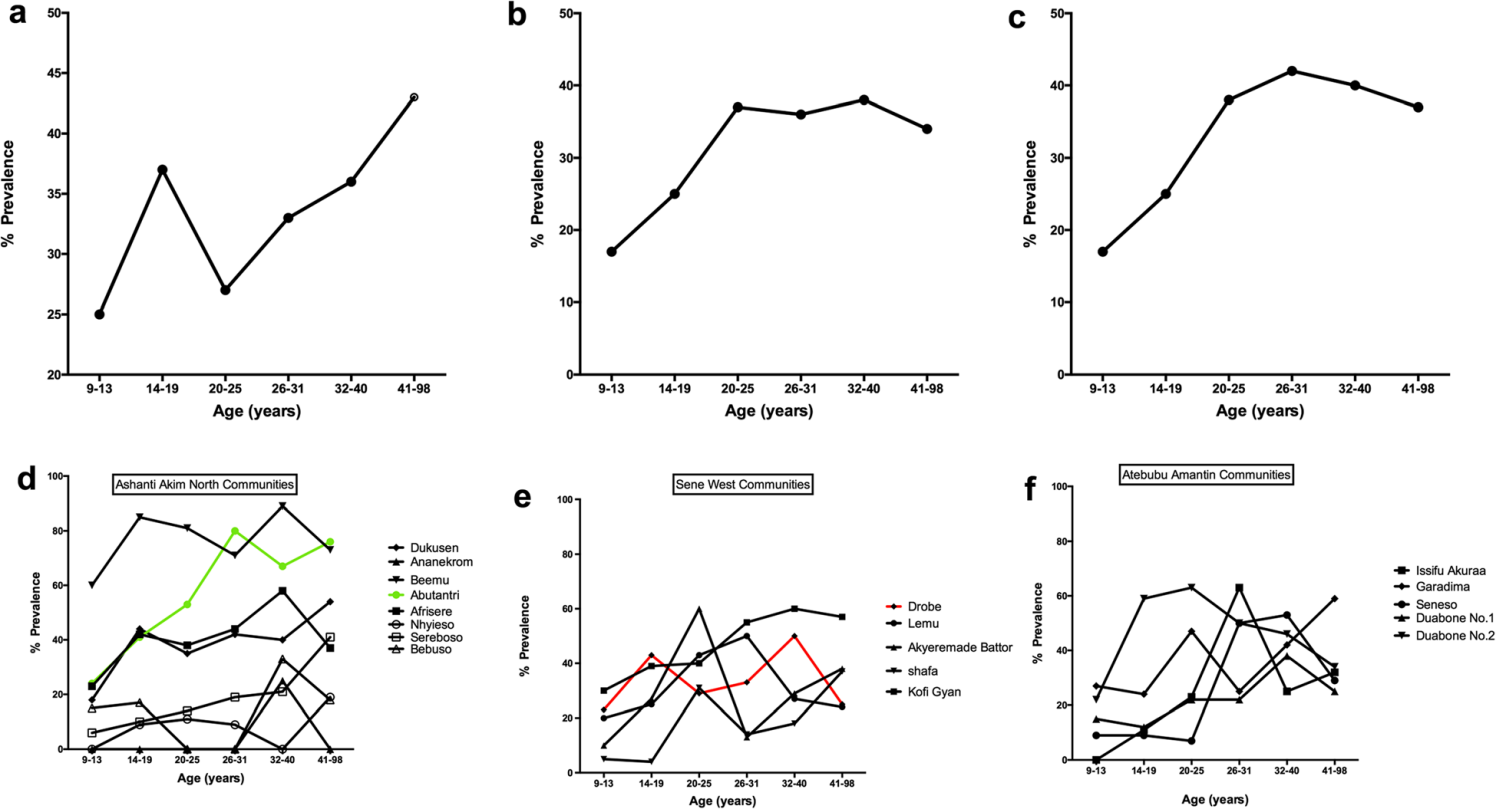
Distribution of *M. perstans* microfilariae within age groups for Ashanti Akim North District (**a**), Sene West District (**b**), Atebubu Amantin District (**c**), as well as distribution within specific communities for Ashanti Akim North District (**d**), Sene West District (**e**) and Atebubu Amantin District (**f**). There were associations between microfilarial prevalence and age in the Ashanti Akim North District (*χ* ^2^ = 24.3, *df* = 8, *P* = 0.0020; Fig. 2a), Sene West District (*χ* ^2^ = 25.1, *df* = 8, *P* = 0.0015; Fig. 2b) and Atebubu Amantin District (*χ* ^2^ = 28.3, *df* = 8, *P* = 0.0004; Fig. 2c). *Green* line depicts the community with co-infections of *Wuchereria bancrofti*. *Red* line shows the community with ongoing Mass Drug Administration Programmes

There was statistical significance in microfilarial prevalence between males and females, with the exception of participants from Sene West District. In all three districts, there were associations between the *M. perstans* infection prevalence and age of study participants (*χ* ^2^ = 24.3, *df* = 8, *P* = 0.0020; *χ* ^2^ = 25.1, *df* = 8, *P =* 0.0015; and *χ* ^2^ = 28.3, *df* = 8, *P* = 0.0004, respectively). In the Ashanti Akim North District the prevalence increased with age with an exceptionally high prevalence within the 14–19 years age group (Fig. 2a). In Sene West and Atebubu Amantin Districts (Fig. 2b, c) microfilarial prevalence increased from age group 9–13 years until it plateaued from age 20–25 years.

Ashanti Akim North District had the highest overall prevalence of 33% with some communities recording over 70% microfilarial prevalence. In Sene West and Atebubu Amantin Districts the prevalence rates were similar to those of Ashanti Akim North District at 31% (Table 1). Within the various districts the prevalence differed from one community to the other. Communities in the Ashanti Akim North District showed the highest variation with 2% in Ananekrom and 75% in Beemu (Additional file 1: Table S1; Fig. 2d). Sampled areas in the Atebubu Amantin and Sene West Districts were more comparable with *M. perstans* prevalence ranging from 22–41% and 18–46%, respectively (Additional file 1: Table S1; Fig. 2e, f).

Further analysis was carried out for Drobe, a community where participants had received Ivermectin for the treatment of onchocerciasis for more than five years under Mass Drug Administration (MDA) program. MDA had taken place 3 months prior to the present study. From 107 patients screened in that community, 58 (54.2%) had taken part in MDA and of those, 35 individuals were *M. perstans* MF positive. Twenty of 35 (60%) individuals had taken part in MDA for more than three years. As this study did not include MDA treatment as a factor, the impact of MDA on *M. perstans* infection was not evaluated in detail.

Co-infection with *Wuchereria bancrofti* MF was co-incidentally identified in five people from Abutantri community in the Ashanti Akim North District. The MF were differentiated by their size and presence of sheath around the MF.

### Entomological findings

From June 2014 to October 2015 an entomological survey to identify diversity of *Culicoides* species in Ashanti Akim North District was undertaken. A total of 2,207 *Culicoides* species were caught in eight communities in the Ashanti Akim North District in the communities where the screening of volunteers for *M. perstans* was also performed. *Culicoides* species were identified by wing morphology as previously described by Boormann & Glick [19, 20]. Results presented are for the evening (16:00–17:00 h) and overnight (18:00–6:00 h) collections since no *Culicoides* species was caught in the morning either by light trap or HLC method.

In the dry season, 546 *Culicoides* were collected with the CDC light traps and 364 by the HLC method. The overall *Culicoides* species caught in the rainy season was higher (1,297), with 737 flies caught by the CDC light traps and 560 using the HLC method. The evening catch (16:00–17:00 h) for the wet and dry seasons yielded 833 and 631, respectively, whilst the overnight *Culicoides* collection (18:00–6:00 h) yielded 464 and 279 for the wet and dry season respectively (Additional file 2: Table S2 and Additional file 3: Table S3).

**Fig. 3:**
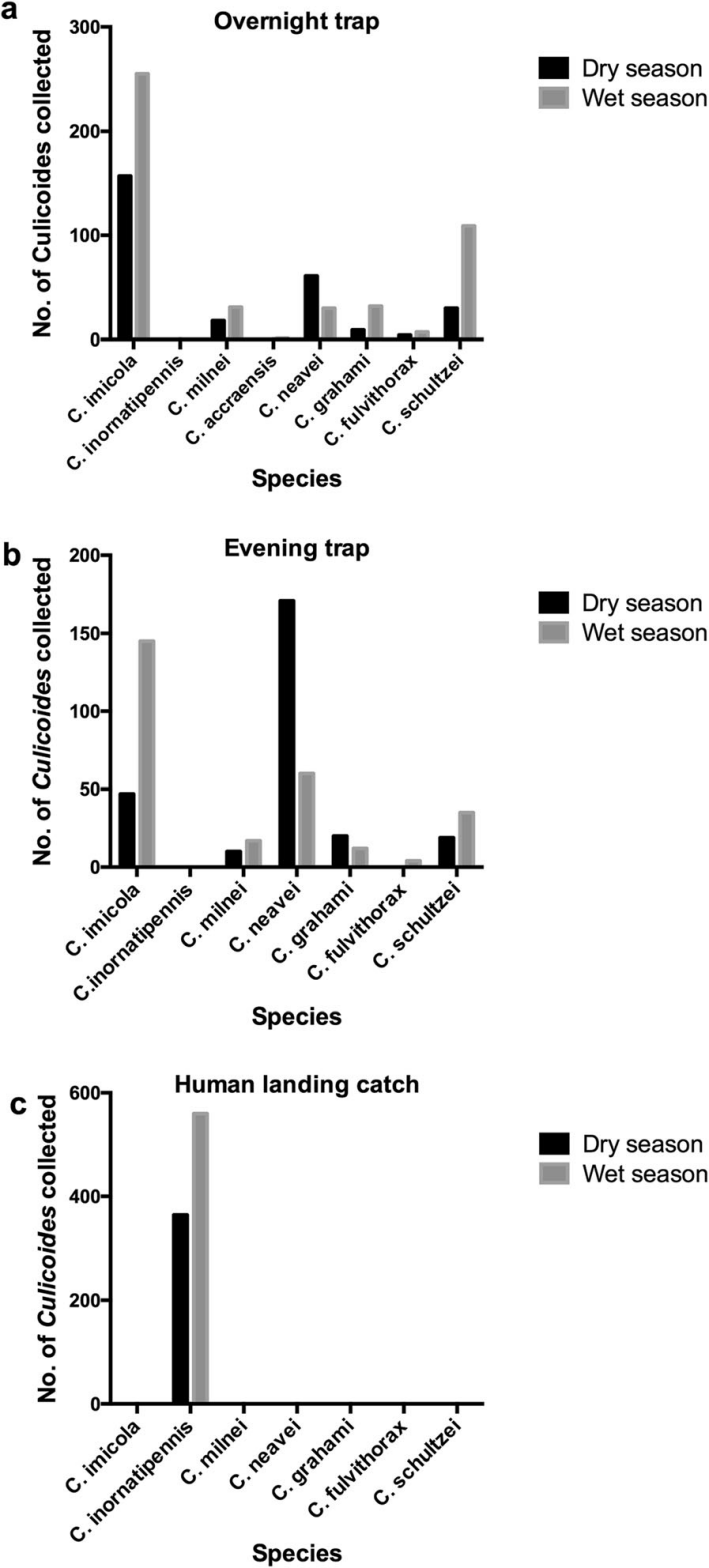
Overnight trap collection (**a**), evening trap collections (**b**) and human landing catch collections (**c**) Comparison of different *Culicoides* species obtained using light traps and human landing catch collections within the dry (black bars) or wet season (grey bars)

Interestingly, *C. inornatipennis* was the only species collected when the HLC method was used irrespective of seasons (wet and dry) or collection times (evening and overnight). This species was recorded in all the communities where the screening was done but was never present in the light trap collections. Figure 3 shows that there was some species diversity in the total trap collection with the highest recorded *Culicoides* species being *C. imicola* (47%) followed by *C. neavei* (25%) then *C. schultzei* (15%) in both seasons at all times. Less than 6% of all the *Culicoides* collected in the evening and overnight collections belonged to other *Culicoides* species (Additional file 2: Table S2 and Additional file 3: Table S3).

These species were also not present in the HLC method. Although the total number of *Culicoides* collected were higher in the wet season when compared with the dry season the species diversity was comparable. A non-parametric correlation analysis revealed a significant correlation between *M. perstans* prevalence and *C. inornatipennis* only in the dry season (Table 2). But there was no correlation among any of the other *Culicoides* species regardless of the season.

**Table 2:** Correlation between *M. perstans* prevalence and *Culicoides* spp. abundance: evening trap and human landing catch collections

		*C. inornatipennis*	*C. milnei*	*C. grahami*	*C. neavei*	*C. imicola*
Dry season	*r*	0.847*	0.275	0.408	-0.144	-0.234
	*P*	0.016	0.55	0.364	0.758	0.613
Wet season	*r*	0.286	-0.546	-0.179	-0.577	-0.214
	*P*	0.535	0.205	0.701	0.175	0.645

*Signifcant at *P* < 0.05

## Discussion

Even though *M. perstans* was suspected to be present in Ghana (Awadzi et al. [14]), verification was only ascertained when MF were coincidentally found in stained peripheral blood mononuclear cells (PBMCs) from patients with Buruli ulcer [15]. The overall prevalence of *M. perstans* in this study was found to be 32%, with a wide variation between 2 and 75% (Additional file 1: Table S1). This is understandable since *M. perstans* distribution is known to vary from areas of low endemicity to areas where almost everyone is infected [11, 21, 22]. This prevalence rate is high compared with several areas in which this infection has been reported [2, 6, 23, 24] even though higher prevalence rates of over 50% have also been reported in other studies [6, 25–27].

Generally, the prevalence of *M. perstans* infection was dependent on host gender since our survey indicated that males had a higher chance of acquiring the infection than females probably because males are more exposed than females. However, in the Sene West District, males and females appeared to have equal chances of acquiring the infection. This is in conformity with previous gender associations [7, 11] but others could not confirm this [6, 28, 29].

There have also been suggestions that age influences *M. perstans* infection. It is noteworthy that in our study, young volunteers from nine years of age were infected with *M. perstans* and the rate of infection increased with age as was observed elsewhere [3, 6, 23]. The 20 to 45-year-old volunteer group had a higher chance of being infected when compared to those in other age groups, probably because they are more active and therefore more likely to be exposed to vector bites. It is possible that the cumulative effect of reinfection plays a role in adults getting infected than children. In low transmission communities the differences in MF counts between adults and children suggested a slow steady increase with age [30] whereas high transmission areas had a more rapid increase with age [6]. There was a progressive rise in prevalence with age irrespective of high transmission or low transmission [6].

The selected communities in this study were surrounded by vegetation (plantain farms) and livestock that are known to be breeding grounds for biting midges but the community with the highest prevalence (> 50%) had plantain vegetation that was more dense or swampy with a lot of compost material from the decaying of the plantain stems and droppings from the livestock. Probably that might be the preferred breeding ground for the midges as was observed in other studies [1, 16, 31].

The wide distribution of *M. perstans* MF in the districts suggests a broad distribution of the vector in the Ashanti Akim District. The communities with the high microfilarial prevalence were also noted to have high distribution of *C. innonatipennis* collected with the HLC method. Although previously *C. austeni* (currently *C. milnei*), *C. grahami* and *C. fulvithorax* were noted for transmission of the parasite [16–18, 31] their proportion in this study was less than 6%, and there was no significant correlation with the prevalence of *M. perstans* in the dry and wet seasons. The vegetation in *M. perstans* endemic communities comprised of plantain farms, shrubs and decaying plant matters which are known to favour the breeding of *Culicoides* [19].

Trap collections gave seven different species (*C. grahami*, *C. milnei*, *C. neavei*, *C. imicola*, *C. fulvithorax*, *C. schultzei* and *C. accraensis*) whereas HLC gave only one species (*C. inornatipennis*). This suggests that of all the collected species, only *C. inornatipennis* was preferentially anthropophilic. Similarly, this finding is in consonance with those of Viennet et al. [32] who collected a higher diversity of species (15 species) using the UV light trap compared to direct aspiration.

## Conclusion

To our knowledge, this is the first study investigating the prevalence of mansonellosis in Ghana in a large study population. Our study proves the presence of mansonellosis in Ghana with over 30% of the people in the study communities being infected. This study therefore provides a platform for additional investigations into the relevance of this filarial, especially its role in co-infections. Interestingly, this study also revealed that the diversity of *Culicoides*, obtained in an endemic area, depends on the type of trap. Here we show that the human landing catch method collected only one species (*C. inornatipennis*) whereas the CDC light traps caught seven different species (*C. grahami*, *C. milnei*, *C. neavei*, *C. imicola*, *C. fulvithorax*, *C. schultzei* and *C. accraensis*) but not *C. inornatipennis*. Such findings need to be taken into consideration when performing entomological studies on *Culicoides* species. The species responsible for transmitting *M. perstans* in Ghana is likely to be *C. inornatipennis*, but formal proof has yet to be obtained.

## Additional files

Additional file 1: Table S1. Demographic sistribution of volunteers in study communities. (DOCX 83 kb)
Additional file 2: Table S2. Evening trap and hlc collections (4–7 pm) for dry season and wet season. (DOCX 106 kb)
Additional file 3: Table S3. Overnight trap collections for dry season and wet season (6 pm – 6 am). (DOCX 67 kb)

## Abbreviations

CDC: Centre for Disease Control
MDA: Mass drug administration
LTC: Light trap collection
HLC: Human landing catch
PBMCs: Peripheral blood mononuclear cells
