# Supplementary material for: Epidemiology of Mansonella perstans in the middle belt of Ghana
Source: Parasit Vectors. 2017 Jan 7;10:15. doi: 10.1186/s13071-016-1960-0 (PMC5219801; doi:10.1186/s13071-016-1960-0)
Supplement: Additional file 1: Table S1. — Demographic sistribution of volunteers in study communities. (DOCX 83 kb) [file 13071_2016_1960_MOESM1_ESM.docx]

Additional file 1: Table S1. Demographic sistribution of volunteers in study communities

|  | Communities | No. screened | Mean age (range) | Male/Female | MF prevalence |
| --- | --- | --- | --- | --- | --- |
| Ashanti Akim North district | 1 | 199 | 28.0 (9–80) | 99/100 | 19 |
|  | 2 | 192 | 31.4 (10–70) | 87/105 | 13 |
|  | 3 | 116 | 30.8 (9–79) | 67/49 | 40 |
|  | 4 | 203 | 25.2 (9–72) | 108/95 | 75 |
|  | 5 | 93 | 28.7 (9–72) | 49/44 | 14 |
|  | 6 | 119 | 25.9 (9–70) | 46/73 | 2 |
|  | 7 | 163 | 25.4 (9–65) | 74/89 | 38 |
|  | 8 | 130 | 28.5 (9–98) | 71/59 | 54 |
| Atebutu Amantin district | 9 | 100 | 30.8 (10–89) | 48/52 | 22 |
|  | 10 | 100 | 31.3 (9–80) | 51/49 | 41 |
|  | 11 | 109 | 28.8 (9–86) | 54/55 | 39 |
|  | 12 | 99 | 32.5 (9–85) | 49/50 | 23 |
|  | 13 | 100 | 30.2 (9–85) | 51/49 | 30 |
| Sene West district | 14 | 100 | 31.1 (9–85) | 61/39 | 26 |
|  | 15 | 107 | 32.8 (9–91) | 42/65 | 33 |
|  | 16 | 100 | 29.2 (9–80) | 64/36 | 46 |
|  | 17 | 113 | 34.7 (9–80) | 39/74 | 30 |
|  | 18 | 104 | 30.2 (9–92) | 45/59 | 18 |
| Total |  | 2247 | 29.4(9–98) | 1105/1142 | 32 |

*Abbreviations*: 1, Sereboso; 2, Nhyieso; 3, Dukusen; 4, Beemu; 5, Bebuso; 6, Ananekrom; 7, Afrisere; 8, Abutantri; 9, Duabone N0.1; 10, Duabone N0. 2; 11, Garadima; 12, Issifu Akuraa; 13, Seneso; 14, Akyeremade Battor; 15, Drobe; 16; Kofi Gyan; 17, Lemu; 18, Shafa
