# Supplementary material for: Epidemiology of Mansonella perstans in the middle belt of Ghana
Source: Parasit Vectors. 2017 Jan 7;10:15. doi: 10.1186/s13071-016-1960-0 (PMC5219801; doi:10.1186/s13071-016-1960-0)
Supplement: Additional file 2: Table S2. — Evening trap and hlc collections (4–7 pm) for dry season and wet season. (DOCX 106 kb) [file 13071_2016_1960_MOESM2_ESM.docx]

Additional file 2:Table S2. Evening trap and hlc collections (4–7 pm) for dry season and wet season

|  |  | Communities | | | | | | | | | | | | | | Total |
| --- | --- | --- | --- | --- | --- | --- | --- | --- | --- | --- | --- | --- | --- | --- | --- | --- |
|  | **Species** | **Serebuoso** | | **Afrisere** | | **Anokye-beemu** | | **Abutantri** | | **Dukusen** | | **Bebuso** | | **Nhyieso** | |  |
|  |  | **trap** | **hlc** | **trap** | **hlc** | **trap** | **hlc** | **trap** | **hlc** | **trap** | **hlc** | **trap** | **hlc** | **trap** | **hlc** |  |
| Dry season | *C. imicola* | 10 | 0 | 6 | 0 | 0 | 0 | 1 | 0 | 22 | 0 | 0 | 0 | 8 | 0 | 47 |
|  | *C.inornatipennis* | 0 | 4 | 0 | 4 | 0 | 89 | 0 | 213 | 0 | 46 | 0 | 6 | 0 | 2 | 364 |
|  | *C. milnei* | 2 | 0 | 1 | 0 | 0 | 0 | 3 | 0 | 3 | 0 | 1 | 0 | 0 | 0 | 10 |
|  | *C. neavei* | 8 | 0 | 22 | 0 | 5 | 0 | 21 | 0 | 73 | 0 | 34 | 0 | 8 | 0 | 171 |
|  | *C. grahami* | 2 | 0 | 1 | 0 | 0 | 0 | 14 | 0 | 3 | 0 | 0 | 0 | 0 | 0 | 20 |
|  | *C. schultzei* | 5 | 0 | 0 | 0 | 0 | 0 | 0 | 0 | 6 | 0 | 5 | 0 | 3 | 0 | 19 |
|  | **Total** | **27** | **4** | **30** | **4** | **5** | **89** | **39** | **213** | **107** | **46** | **40** | **6** | **19** | **2** | 631 |
| Wet season | *C. imicola* | 5 | 0 | 3 | 0 | 0 | 0 | 9 | 0 | 110 | 0 | 2 | 0 | 16 | 0 | 145 |
|  | *C.inornatipennis* | 0 | 7 | 0 | 11 | 0 | 100 | 0 | 58 | 0 | 54 | 0 | 294 | 0 | 36 | 560 |
|  | *C. milnei* | 0 | 0 | 1 | 0 | 1 | 0 | 0 | 0 | 2 | 0 | 9 | 0 | 4 | 0 | 17 |
|  | *C. neavei* | 10 | 0 | 3 | 0 | 2 | 0 | 14 | 0 | 2 | 0 | 9 | 0 | 20 | 0 | 60 |
|  | *C. grahami* | 0 | 0 | 0 | 0 | 0 | 0 | 2 | 0 | 2 | 0 | 0 | 0 | 8 | 0 | 12 |
|  | *C. fulvithorax* | 0 | 0 | 0 | 0 | 0 | 0 | 0 | 0 | 0 | 0 | 2 | 0 | 2 | 0 | 4 |
|  | *C. schultzei* | 2 | 0 | 1 | 0 | 0 | 0 | 0 | 0 | 4 | 0 | 2 | 0 | 26 | 0 | 35 |
|  | Total | 17 | 7 | 8 | 11 | 3 | 100 | 25 | 58 | 120 | 54 | 24 | 294 | 76 | 36 | 833 |

*Abbreviation*: hlc, human landing catches

*Note*: Dry season: November to February; Wet season: June to October
