# Supplementary material for: Epidemiology of Mansonella perstans in the middle belt of Ghana
Source: Parasit Vectors. 2017 Jan 7;10:15. doi: 10.1186/s13071-016-1960-0 (PMC5219801; doi:10.1186/s13071-016-1960-0)
Supplement: Additional file 3: Table S3. — Overnight trap collections for dry season and wet season (6 pm – 6 am). (DOCX 67 kb) [file 13071_2016_1960_MOESM3_ESM.docx]

Additional file 3: Table S3. Overnight trap collections for dry season and wet season (6 pm – 6 am)

|  | **Communities – dry season/wet season** | | | | | | |  |
| --- | --- | --- | --- | --- | --- | --- | --- | --- |
| **Species** | **Serebuoso** | **Afrisere** | **Anokye-beemu** | **Abutantri** | **Dukusen** | **Bebusu** | **Nhyieso** | **Total** |
| *C. imicola* | 0/2 | 6/2 | 1/3 | 2/14 | 139/225 | 1/3 | 8/6 | **157/255** |
| *C. inornatipennis* | 0/0 | 0/0 | 0/0 | 0/0 | 0/0 | 0/0 | 0/0 | **0/0** |
| *C. milnei* | 0/0 | 0/1 | 2/2 | 6/4 | 7/5 | 2/5 | 1/14 | **18/31** |
| *C. accraensis* | 0/1 | 0/0 | 0/0 | 0/0 | 0/0 | 0/0 | 0/0 | **0/1** |
| *C. neavei* | 2/9 | 0/1 | 9/0 | 18/10 | 17/1 | 14/6 | 1/3 | **61/30** |
| *C. grahami* | 0/2 | 0/0 | 0/0 | 2/22 | 5/6 | 0/0 | 2/2 | **9/32** |
| *C. fulvithorax* | 0/0 | 0/0 | 1/1 | 0/0 | 0/0 | 2/2 | 1/3 | **4/7** |
| *C. schultzei* | 0/0 | 0/2 | 0/0 | 1/0 | 15/4 | 12/6 | 2/97 | **30/109** |
| **Total** | **2/14** | **6/6** | **13/6** | **29/50** | **183/241** | **31/22** | **15/125** | **279/464** |

*Note*: Dry season, November to February; Wet season, June to October
